# Targeting IS*608* transposon integration to highly specific sequences by structure-based transposon engineering

**DOI:** 10.1093/nar/gky235

**Published:** 2018-04-09

**Authors:** Natalia Rosalía Morero, Cecilia Zuliani, Banushree Kumar, Aleksandra Bebel, Sachi Okamoto, Catherine Guynet, Alison Burgess Hickman, Michael Chandler, Fred Dyda, Orsolya Barabas

**Affiliations:** 1 Structural and Computational Biology Unit, European Molecular Biology Laboratory, Heidelberg 69117, Germany; 2 Laboratoire de Microbiologie et Génétique Moléculaires, Centre National de la Recherche Scientifique, Toulouse Cedex 31062, France; 3 Laboratory of Molecular Biology, National Institute of Diabetes and Digestive and Kidney Diseases, National Institutes of Health, Bethesda, MD 20892, USA

## Abstract

Transposable elements are efficient DNA carriers and thus important tools for transgenesis and insertional mutagenesis. However, their poor target sequence specificity constitutes an important limitation for site-directed applications. The insertion sequence IS*608* from *Helicobacter pylori* recognizes a specific tetranucleotide sequence by base pairing, and its target choice can be re-programmed by changes in the transposon DNA. Here, we present the crystal structure of the IS*608* target capture complex in an active conformation, providing a complete picture of the molecular interactions between transposon and target DNA prior to integration. Based on this, we engineered IS*608* variants to direct their integration specifically to various 12/17-nt long target sites by extending the base pair interaction network between the transposon and the target DNA. We demonstrate *in vitro* that the engineered transposons efficiently select their intended target sites. Our data further elucidate how the distinct secondary structure of the single-stranded transposon intermediate prevents extended target specificity in the wild-type transposon, allowing it to move between diverse genomic sites. Our strategy enables efficient targeting of unique DNA sequences with high specificity in an easily programmable manner, opening possibilities for the use of the IS*608* system for site-specific gene insertions.

## INTRODUCTION

Transposable elements (TEs) are a large, ubiquitous group of mobile genetic elements that can autonomously move from one genomic location to another. They have had a dynamic role in genome remodelling and evolution, and most eukaryotic and prokaryotic genomes are rich in TE-related sequences (1–4). While most of these represent inactive TE remnants, various elements can still move, causing diverse adaptive or adverse phenotypes throughout the tree-of-life (5,6). For example, in bacteria TE mobilization has been linked to environmental adaptation and the emergence of multi-drug resistant pathogens (7,8). Due to their inherent ability to carry and integrate DNA into foreign genomes, TEs provide widely used tools for genetic engineering. They have been successfully used for insertional mutagenesis allowing for example the characterization of gene functions and the identification of oncogenes and tumour suppressors (9). Moreover, TEs that move exclusively using DNA intermediates (DNA transposons) are also applied in transgenesis, providing efficient non-viral gene delivery vehicles that are now used in human gene therapy applications (10). However, a major constraint of these tools in transgenesis is the very low specificity of their target site selection (e.g. the most used *Sleeping Beauty* and *PiggyBac* transposons integrate at specific di- or tetranucleotide sequences, respectively), which leads to integration at diverse positions throughout the recipient genome. Therefore, much effort has been dedicated to unravel the molecular basis of target DNA selection and transposition of a variety of TEs, in order to optimize TE-based genetic tools and to design strategies to direct their integration to specific genomic sites.

**Figure 1.**
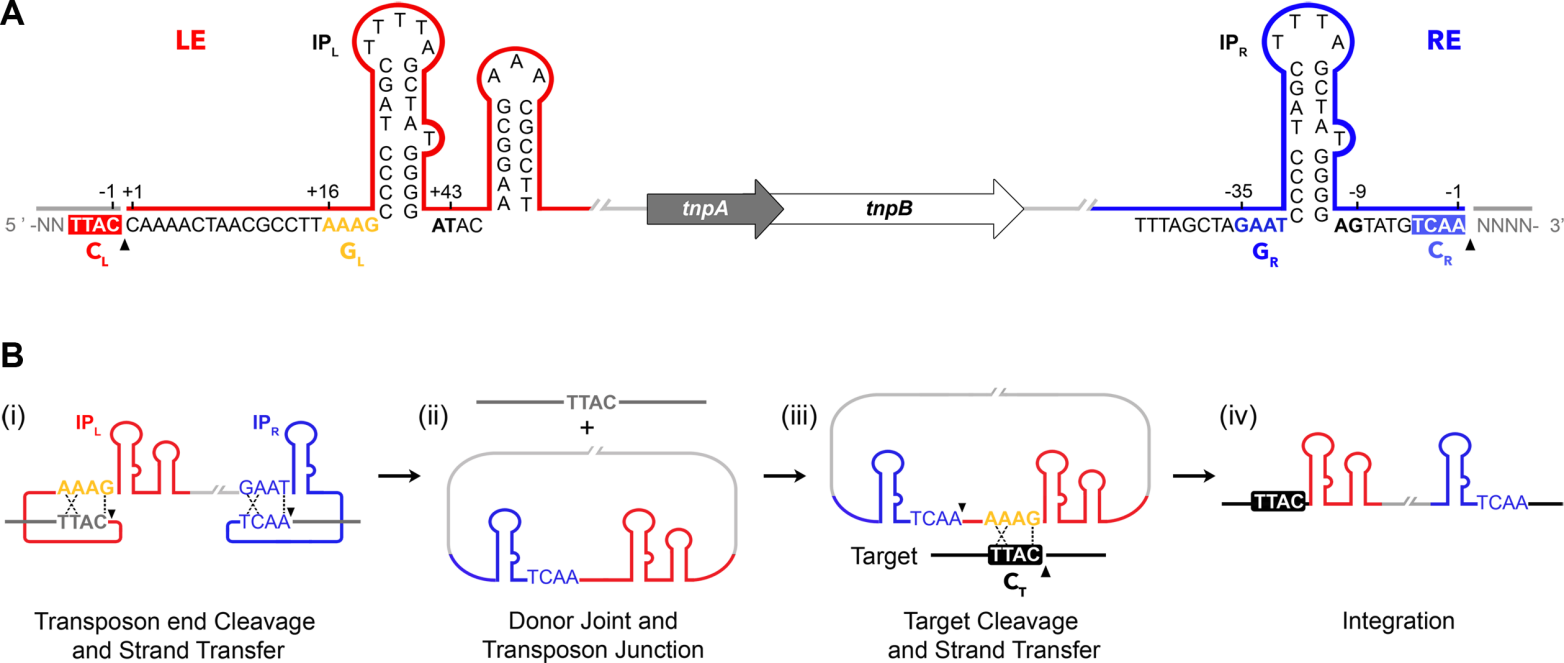
The IS*608* structure and transposition mechanism (adapted from (16)). (**A**) The IS*608* left (LE, red) and right (RE, blue) ends flank the *tnpA* and *tnpB* open reading frames (block arrows). Subterminal imperfect palindromes (IP_R_ and IP_L_), right and left cleavage sites (C_R_: TCAA and C_L_: TTAC) and right and left guide sequences (G_R_: GAAT and G_L_: AAAG) are highlighted. Black wedges mark the positions of cleavage and 5′ phosphotyrosine TnpA-DNA intermediate formation. (**B**) Model of the IS*608* transposition pathway. After transposon end cleavage (i), the donor DNA sequence is precisely sealed and a circular transposon junction is formed (ii) as an intermediate before cleavage and re-integration into a new target site (iii and iv). Black wedges mark the positions of cleavage at the transposon ends (i) and 3′ to a target cleavage site (C_T_) (iii). Specific base-pairing between guide and cleavage sequences in the transposon before excision (i), and between G_L_ and C_T_ before re-integration (iii), are indicated with dotted lines.

One of the simplest and best characterized TEs is the bacterial insertion sequence (IS) IS*608* from *Helicobacter pylori*, a member of the IS*200*/IS*605* family (11). It exhibits an unusual transposition mechanism using single-stranded DNA (ssDNA) intermediates and integrates specifically at 4 nt target sequences in the genome (12). Transposon excision and integration is catalyzed by the IS*608*-encoded transposase TnpA (Figure 1A), which belongs to the HUH (histidine–hydrophobic–histidine) endonuclease superfamily and uses a single catalytic tyrosine to cleave DNA. Previous crystallographic and biochemical studies of IS*608* have shown that TnpA binds specifically to sub-terminal imperfect palindrome (IP) structures formed on the top strand of the left (LE) and right (RE) IS ends (named IP_L_ and IP_R_, respectively) (Figure 1A) (13). Its catalytic site is assembled in *trans* within a protein dimer, with the catalytic tyrosine (located on the most C-terminal helix αD) contributed by one monomer and the HUH motif by the other (13). IS*608* insertion occurs precisely 3′ to a specific TTAC tetranucleotide sequence that is then retained in the left transposon flank and is required for excision and subsequent transposition to a new site. Notably, the cleavage site sequences at LE and RE (C_L_ and C_R_, respectively) are not directly recognized by TnpA, but form a complex set of base pairs with a tetranucleotide ‘guide’ sequence (G_L_ or G_R_) located 5′ to the base of each IP hairpin (Figure 1B) (14,15). These interactions help to structure the nucleoprotein complex and activate transposon excision, creating a circular junction intermediate and simultaneously sealing the flanking donor DNA backbone. For integration, the TnpA-bound transposon junction specifically interacts with an ssDNA target by base pairing between G_L_ and the TTAC target sequence (C_T_, Figure 1B). This unique mode of target recognition by base pairing between transposon and target sequences provides an intriguing opportunity to redirect transposon integration to different sequences in a predictable way by only modifying G_L_ in the transposon, as demonstrated previously *in vitro* and *in vivo* (16).

In the present work, we aimed to expand the network of transposon-target DNA base pairing to increase the specificity of target recognition and direct IS*608* integration to longer DNA sequences that may be unique in the context of a genome. We present a crystal structure of IS*608* TnpA in a ternary complex with transposon left end DNA (LE29, including IP_L_ with G_L_ and 3 additional nucleotides downstream of IP_L_) and a target substrate (T6’) spanning the cleavage site (C_T_). The structure reveals the IS*608* target capture complex in an active pre-cleavage state with uncleaved target DNA and provides novel insights into the recognition of the nucleotides surrounding the core 4 nt target sequence. It shows that target recognition involves base triplet interactions between G_L_, the 3′ flank of IP_L_ and the target sequence. Based on the structural insights, we design novel transposon variants that create an extended set of specific base interactions with the target DNA, thereby recognizing longer target sites with high specificity. We demonstrate efficient selection of several representative 12 and 17 nt sequences, providing a novel strategy and proof-of-concept for targeting specific user-defined DNA sites.

## MATERIALS AND METHODS

### Oligonucleotides

All oligonucleotides used in this work were purchased from IDT (Coralville, IA). Sequences of oligonucleotides used for crystallization and *in vitro* activity assays are shown in Supplementary Table S1 and sequences of target DNA used in covalent complex formation assays are shown in Figure 3. For hairpin formation in oligonucleotides containing inverted repeat sequences, these were resuspended in TE buffer, heated to 95°C for 10 min, rapidly cooled on ice and placed at –20°C until further use. Where indicated, oligonucleotides were labeled at the 5′-end using [γ-^32^P]-ATP (Hartmann Analytic) and T4 polynucleotide kinase (NEB Inc.).

### Protein purification and crystallization

IS*608* TnpA was purified as previously described (13). TnpA/LE29/T6’ complexes were formed by mixing protein with LE29 and T6’ DNA oligonucleotides in 1:1:1.3 molar ratio at 5 mg/ml final protein concentration, and dialysing against buffer 1 (20 mM Tris–HCl [pH 7.5], 0.5 M NaCl, 0.2 mM TCEP, 2 mM EDTA) and subsequently buffer 2 (20 mM Tris–HCl [pH 7.5], 0.2 M NaCl, 0.2 mM TCEP, 2 mM EDTA). Crystals were obtained by vapour diffusion in hanging drops by mixing complexes 1:1 (v/v) with crystallization buffer (14.6% PEG 3350, 0.19 M calcium acetate). For data collection, crystals were harvested, soaked in cryoprotectant solution (30% PEG 3350, 0.2 M calcium acetate, 30% glycerol) and flash-frozen in liquid nitrogen.

### Data collection and structure determination

Diffraction data for the TnpA/LE29/T6’ crystals were collected on beamline ID30B at the European Synchrotron Radiation Facility (ESRF). Data were processed with XDS (17) and the structure was solved with molecular replacement by Phaser (18), using TnpA/LE26/D6 (PDB ID: 2VJV (14)) as a search model. The obtained structure showed two target capture complexes per asymmetric unit, each of them composed of a TnpA dimer bound to two LE hairpins and two target oligos. The final model was obtained through iterative rounds of manual model building in Coot (19) alternated with cartesian simulated annealing, restrained positional and B factor refinement in Phenix (20). Data collection and refinement statistics are summarized in Table 1. All structure figures were made with PyMOL (Version 1.5.0.4; Schrödinger, LLC).

**Table 1.** Data collection and refinement statistics

	TnpA/LE29/T6′
**Crystal properties**	
Space group	*P*4_1_2_1_2
Unit cell: *a, b, c* (Å)	138.64, 138.64, 117.95
Unit cell: α, β, γ (°)	90, 90, 90
**Data collection**	
Beamline	ID30B (ESRF)
Wavelength (Å)	0.97
Resolution range (Å)	89.84–2.6 (2.691–2.598)
Total reflections	351387 (35340)
Unique reflections	35947 (3525)
Multiplicity	9.8 (10.0)
Completeness (%)	99.72 (99.58)
*R*-merge	0.2929 (1.317)
*R*-meas	0.3091 (1.387)
*R*-pim	0.09723 (0.4295)
*I*/σ*I*	6.03 (1.40)
CC1/2	0.994 (0.738)
Wilson *B*-factor	38.49
**Refinement**	
*R*-work	0.1970 (0.3030)
*R*-free	0.2383 (0.3609)
Number of non-hydrogen atoms	7412
Protein residues	508
RMS (bonds)	0.003
RMS (angles)	0.57
Ramachandran favoured (%)	97.20
Ramachandran outliers (%)	0.00
Clashscore	2.63
Average *B*-factor	34.98
Number of TLS groups	12

Values in parentheses refer to the highest resolution shell.

### DNA cleavage by covalent complex formation

The DNA cleavage activity of TnpA was assayed by covalent complex formation and SDS-PAGE analysis as previously described (14). This assay relies on the formation of a covalent phosphotyrosine bond between TnpA Y127 and the nucleotide flanking the cleavage site upon target cleavage. TnpA/LE complexes (in 1:1.2 molar ratio, with 50 μM TnpA) were prepared on ice and dialyzed against reaction buffer (20 mM Tris–HCl [pH 7.5], 0.2 M NaCl, 20 mM MgCl_2_, 1 mM DTT) at 4°C. Cleavage reactions were set up with 10 μl of the TnpA/LE complex and 60 μM of target DNA (with final TnpA:LE:target complex composition of 1:1.2:1.2 molar ratio). The samples were incubated at room temperature for 1.5 h and then heat-denatured in SDS-containing sample buffer and analyzed by SDS-PAGE. TnpA and TnpA attached to the 16-mer product of target cleavage were detected by Coomassie staining. Novex™ Sharp Pre-stained (Thermo Fisher) protein marker was used as a size standard.

### Oligonucleotide cleavage, strand transfer and integration reactions *in vitro*

Cleavage and strand transfer reactions were based on previously described protocols (21). Briefly, 14 nM of 5′-labeled oligonucleotide (either IS*608* LE, RE, RE-LE junction or target, as indicated) was incubated with 10 μM TnpA for 1 h at 37°C, in buffer containing 20 mM HEPES [pH 7.5], 160 mM NaCl, 5 mM MgCl_2_, 10 mM DTT, 20 μg/ml BSA, 0.5 μg of poly-dIdC and 20% glycerol. For strand transfer reactions, additional unlabeled oligonucleotide substrates were added at 1 μM final concentration. Reactions were terminated by addition of 0.1% SDS and incubation for 15 min at 37°C. Products were heat-denatured, separated on a 10% sequencing TBE-Urea PAGE gel and analyzed by phosphorimaging on a Typhoon™ FLA 9500 (GE Healthcare Life Sciences). A 20/100 Oligo Length Standard (IDT) was radioactively labeled (5′-^32^P) as described above and loaded in every gel.

## RESULTS

### Target recognition in a TnpA/LE29/T6’ pre-cleavage target capture complex

Previous crystal structures of TnpA in complex with IS*608* RE (including G_R_, IP_R_ and C_R_), or with a 26-mer LE (including G_L_ and IP_L_) and a 6-mer target oligo (including the TTAC target site, C_T_), have revealed general principles of transposon end binding (13,14). However, the complete set of interactions involved in target DNA recognition at LE remained unclear, as the 3′ flank of the IP_L_ stem loop, which was predicted to participate in base triplet interactions with G_L_ and target nucleotides (15), was not present in the previous structures. Moreover, the only available IS*608* structure with target DNA included a target substrate ending at the cleavage site (position -1, representing the cleaved product) and crystallized in an inactive conformation, with no metal ion cofactor present and the catalytic tyrosine away from the active site (∼12 Å between Y127/OH and target C^-1^/O3’) (14).

**Figure 2.**
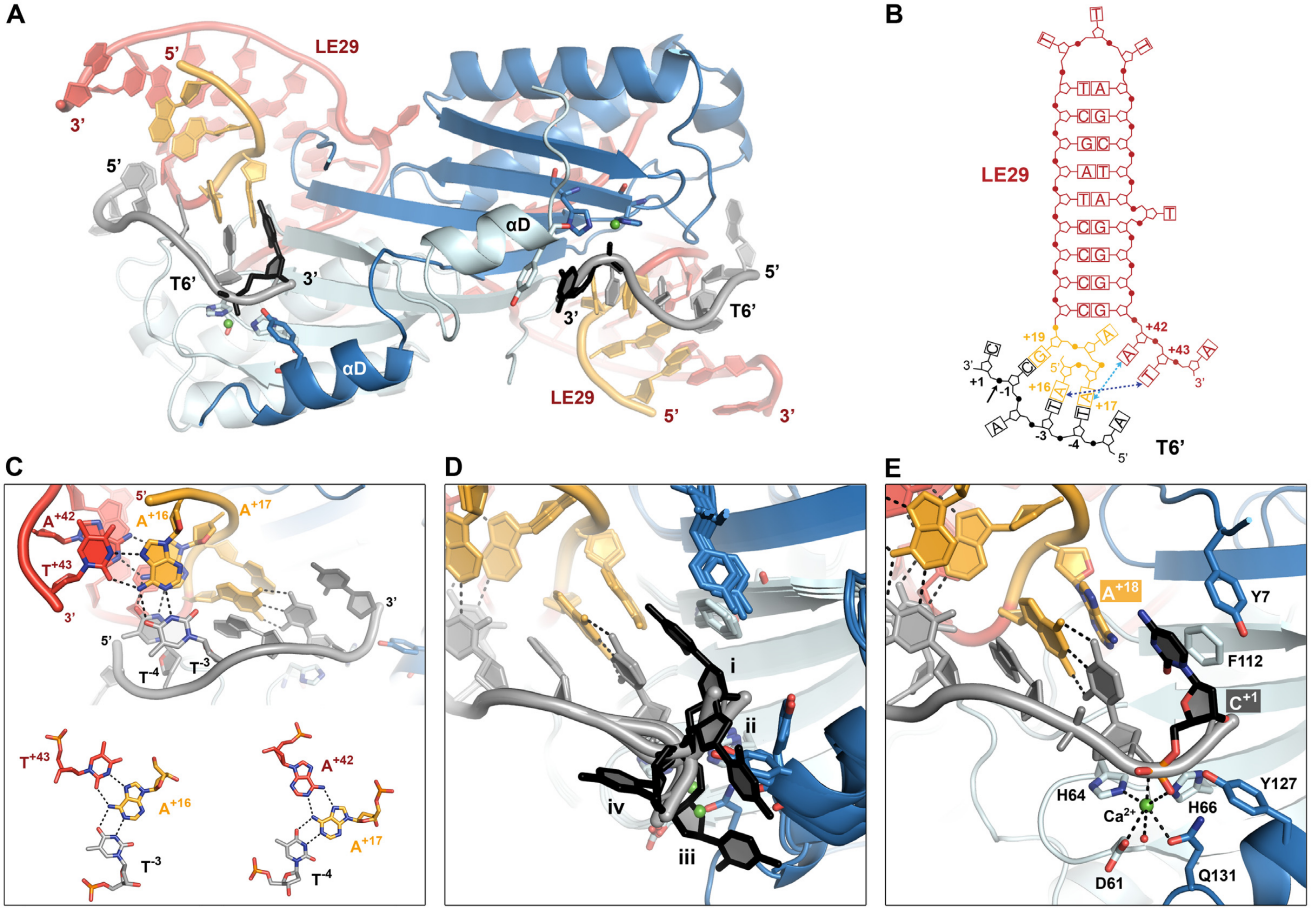
The IS*608* target capture complex structure. (**A**) Overall view of the IS*608* TnpA/LE29/T6’ structure. One of two synaptic complexes in the crystal asymmetric unit is shown. A TnpA dimer (cartoon representation, chain A in light blue and chain B in blue) is bound to two LE29 hairpin DNA molecules (red, G_L_ in orange) and two T6’ target oligos including positions -5 to +1 (grey, with the C^+1^ nucleotide highlighted in black). Catalytic residues are shown in sticks representation with atomic colouring. Ca^2+^ ions are shown as green spheres. (**B**) The architecture of LE29 and its specific base contacts with T6’. Blue dotted arrows indicate non-canonical base interactions between A^+42^ and T^+43^ 3′ of IP_L_ with A^+17^ and A^+16^ from G_L_, respectively, which create base triplets together with T^−4^ and T^−3^ from T6’. (**C**) Two base triplets between LE29 and T6’ (bases in sticks representation with atomic colouring), with hydrogen bonds shown as dotted lines. (**D**) Superposition of the four TnpA/LE29/T6’ complexes (i to iv) present in the crystallographic asymmetric unit, highlighting the different orientations of C^+1^ (black sticks). (**E**) Coordination of the metal ion cofactor in the active site and the position of C^+1^ in the active pre-cleavage conformation (complex i). Catalytic residues as well as amino acids and DNA forming the C^+1^ binding site are shown as sticks with atomic colouring.

In this work, we determined the crystal structure of IS*608* TnpA in complex with a 29-mer LE (LE29) and a 6-mer target oligo (T6’) at 2.6 Å resolution (Table 1, Figure 2 and Supplementary Figure S1). LE29 includes a 3 nt extension at the 3′ base of IP_L_ (positions +42 to +44) and T6’ represents uncleaved target DNA with a cytosine base at position +1 downstream of the cleavage site. The choice of C for this position was based on the observation that most IS*608* integration sites observed *in vivo* in *E. coli* contain a C in position +1 after C_T_ (12). Notably, the LE sequence also contains a C following C_L_ (Figure 1A), indicating a preference for this base. In order to trap the active pre-cleavage state of the complex, crystals with wild type TnpA were grown in the presence of Ca^2+^. This metal has been shown to prevent the enzymatic activity of diverse endonucleases and transposases, including TnpA from the closely related IS*Dra2*, while preserving a high binding affinity and active site geometry (22,23).

The asymmetric unit of the TnpA/LE29/T6’ crystals contains two copies of a ternary complex, each consisting of a TnpA dimer bound to two LE and two target DNA molecules (Figure 2A). The secondary structure of LE29 and specific contacts with T6’ in the complex are represented in Figure 2B. As previously observed in the TnpA/LE26/T6 complex, A^+16^ and A^+17^ in LE29 G_L_ form base pairs with target bases T^−3^ and T^−4^, respectively (14). However, in our structure these pairs also interact with T^+43^ and A^+42^ at the 3′ base of IP_L_, together assembling a set of two base triplets (Figure 2C). These triplet interactions are similar to those observed between G_R_, C_R_ and the 3′ base of IP_R_ in the TnpA/RE35 structure (14) and were proposed to be required also at the LE for stable assembly of synaptic complexes (15).

C^+1^ from T6’ occupies distinct positions in each of the four target molecules present in the crystal asymmetric unit (Figure 2D). Although their locations are well defined in the electron density map, these nucleotides display high B factors, suggesting that they are flexible. In three cases, the C^+1^ base points away from LE29 (Figure 2D, ii–iv) and its position is stabilized by diverse polar contacts on the protein surface, or by π-stacking with Y127. In these cases, the conformation of the swapped helix αD is constrained by crystal contacts, such that it partly unfolds and threads away from the active site (Figure 2A). Consequently Q131, a catalytic residue involved in coordination of the divalent ion cofactor, is in a distant position prohibiting proper metal binding and active site assembly (Supplementary Figure S2A). In one case C^+1^ even occupies the active position of helix αD and blocks the Ca^2+^ binding site. In the fourth T6’ molecule, C^+1^ points towards the A^+18^ base in LE29 (Figure 2D, i) and it is stabilized by π-stacking interactions with Y7 (chain B) and F112 (chain A) (Figure 2E and Supplementary Figure S1). In this case, C^+1^ occupies the position that was occupied by the catalytic Y127 in the previously described TnpA/LE26/T6 structure (14), and helix αD (carrying Y127, chain B) has moved closer to the active site where it is stabilized by hydrophobic contacts and hydrogen bonds involving Q132 and K125 in αD with Q59, E37 and E57 in the protein core, respectively (Supplementary Figure S2A). In this conformation, Y127 is placed in a cleavage-competent position, such that its nucleophile hydroxyl group is positioned 3.0 Å from the phosphorous atom of the scissile phosphate in C^+1^ (Supplementary Figure S1). The Ca^2+^ ion is coordinated by H64 and H66 (β4, chain A), D61 (β3, chain A), Q131 (αD, chain B), C^−1^/O3′ (target DNA), a water molecule and potentially the scissile phosphate (C^+1^/OP1, target DNA) (Figure 2E). This active site conformation and metal ion coordination geometry is very similar to that observed in the post-cleavage TnpA/RE35 complex (14) (Supplementary Figure S2B), indicating that the presence of the scissile phosphate and the first nucleotide flanking the cleavage site does not majorly influence active site geometry.

### TnpA prefers target sites with a C in position +1

**Figure 3.**
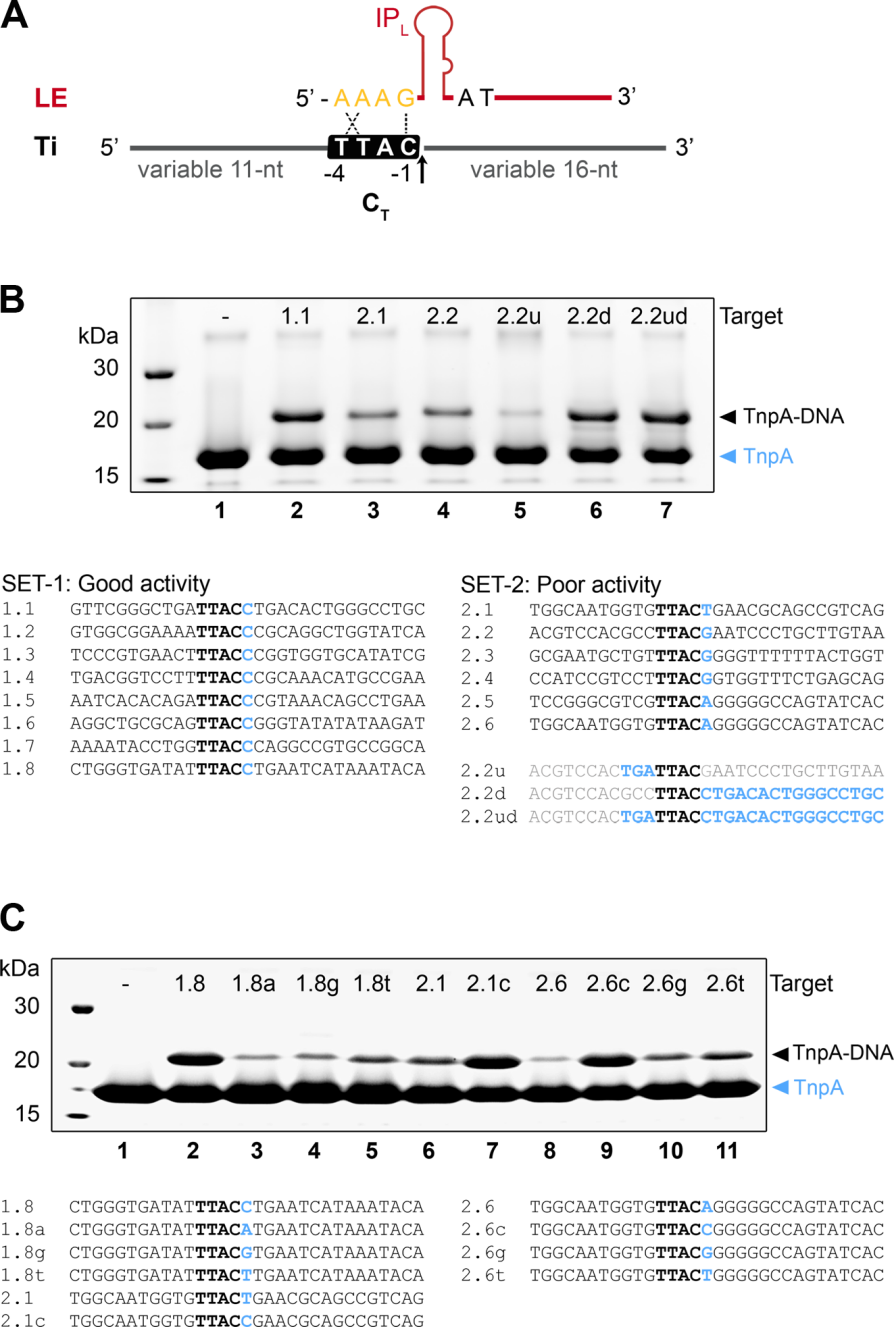
Sequence hallmarks affecting IS*608* target selection. (**A**) Scheme of the IS*608* left end (LE) and target oligos (Ti) used to monitor TnpA mediated cleavage with variable sequences upstream and downstream of the core TTAC target sequence (C_T_). Arrow indicates the position of target cleavage. (**B**) Cleavage assays monitoring covalent TnpA-DNA complex formation on SDS-PAGE gels. Upon Ti cleavage, TnpA becomes covalently attached to the variable 16 nt sequence downstream of the cleavage position and can be resolved from unmodified TnpA. Targets were classified into sets with good (SET-1) and poor activity (SET-2), as shown below the gel. Cleavage reactions are shown for representative SET-1 and SET-2 targets (lanes 2–4). The negative control (lane 1) does not contain target DNA. Cleavage reactions for derivatives of target 2.2, with the sequence upstream (u), downstream (d) of TTAC or both (ud) replaced by the corresponding sequence from target 1.1 (see sequences below SET-2) are shown in lanes 5–7. (**C**) Mutation of the nucleotide C in position +1 compromises cleavage in representative SET-1 targets (lanes 2–5), whereas introduction of a C at this position rescues activity of weak SET-2 targets (lanes 6–11). Covalent complex formation is monitored on SDS-PAGE and target sequences are shown below.

To determine whether the cleavage activity of TnpA is affected by the sequence surrounding the target cleavage site (TTAC, C_T_) and in particular by the identity of the nucleotide in position +1 downstream of the cleavage site, we performed *in vitro* target cleavage assays with target oligonucleotides containing variable sequences at both sides of the TTAC sequence (Figure 3A). For this, TnpA/LE complexes were incubated with different targets, and the cleavage activity was monitored by comparing the ratio of free TnpA and TnpA covalently bound to the 3′ flank of cleaved substrates in SDS-gels (Figure 3B). Based on the levels of covalent complexes formed, targets were classified as: SET-1, with good cleavage activity; and SET-2, with poor activity (Figure 3B, lower panel). Remarkably, SET-1 contained only targets with a C in position +1, whereas SET-2 included other nucleotides. We then replaced the sequence upstream and/or downstream of C_T_ in a SET-2 representative oligo (2.2) with the corresponding sequence from an efficient SET-1 target (1.1) (Figure 3B, lanes 5–7). This showed that the upstream sequence had little effect on cleavage activity, whereas replacement of the sequence downstream of C_T_ greatly increased cleavage, indicating its role in determining cleavage efficiency. To directly test the specific impact of C^+1^ on cleavage activity in particular, we performed gain and loss-of-activity experiments by changing only the nucleotide in this position in target oligos from SET-1 (1.8) and SET-2 (2.1 and 2.6) (Figure 3C). The results revealed that replacing C^+1^ with other nucleotides in a SET-1 target reduced TnpA cleavage activity, whereas introducing a C in position +1 in a SET-2 target rescued cleavage, clearly showing that substrates with C in this position are better targets for TnpA.

### IS*608* target specificity can be increased by rational design of extended base pairing

**Figure 4.**
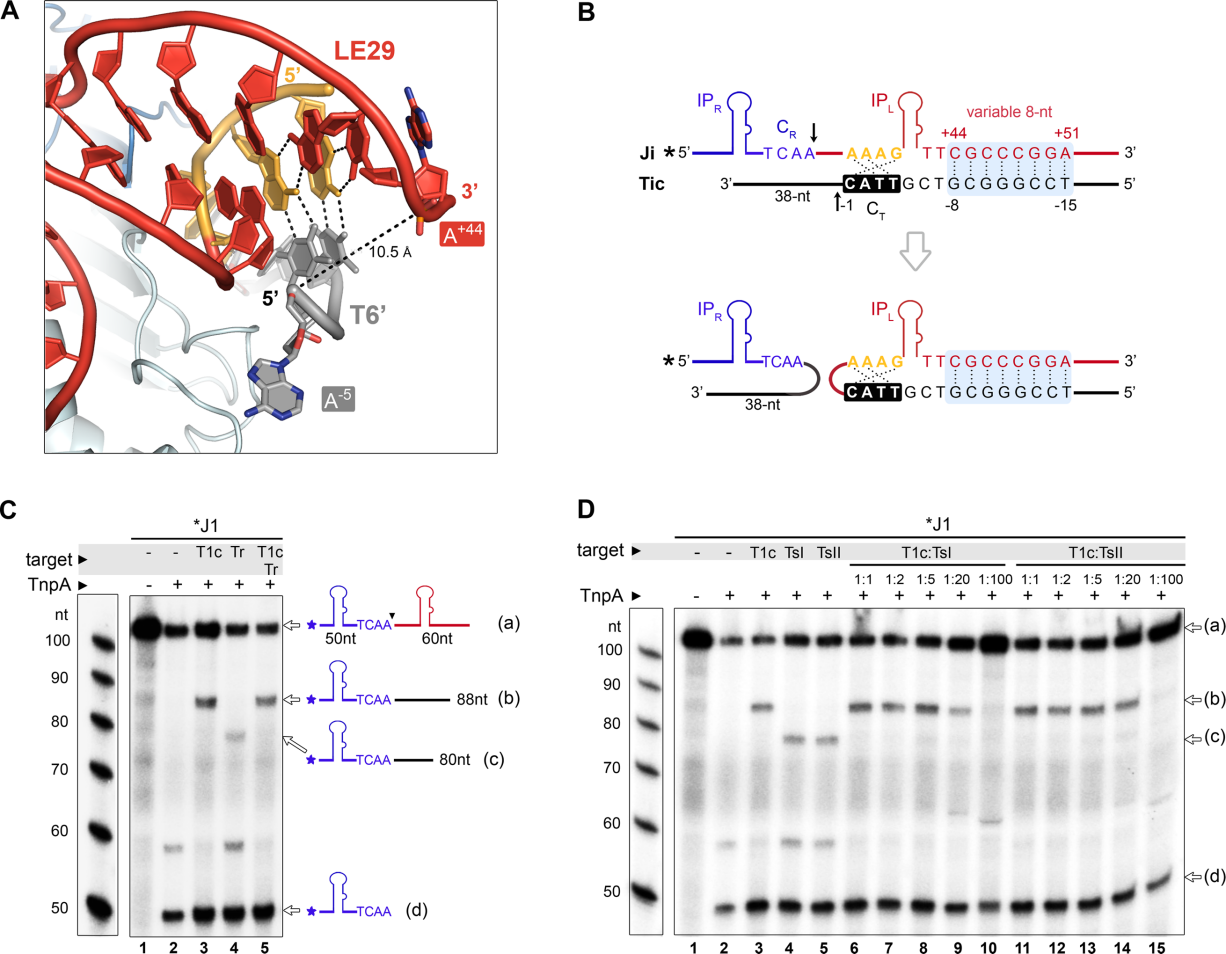
IS*608* can be specifically targeted to longer integration sites by extended LE/target base pairing. (**A**) Close-up of the TnpA/LE29/T6’ structure, highlighting the proximity between the 3′ end of IP_L_ and the 5′ end of the target oligonucleotide. The distance between the O5′ oxygen atom of A^−5^ in T6’ and the phosphorous atom (P) of A^+44^ in LE29 is 10.5 Å (dashed line). (**B**) Design of the IS*608* transposon junction (Ji, where ‘i’ is a variable indicating a specific variant number) and complementary target substrates (Tic, with ‘i’ marking a specific variant as above) used for retargeting. Each set of Ji/Tic oligos was designed to include an 8 bp complementary region between the 3′ extension of the IP_L_ and the sequence upstream of the native TTAC target site (light blue shade). The 8 bp complementary sequence displayed here corresponds to the J1/T1c pair. ^32^P radioisotope labeling is indicated by an asterisk. Upon target cleavage and integration (at the arrow), the radiolabeled 5′ segment of the junction upstream of the cleavage site (50 nt) is attached to the 3′ segment of the target (38 nt). (**C**) Sequencing DNA PAGE gel monitoring J1 cleavage and integration into its T1c complementary target. A random target substrate containing a TTAC site but no additional complementarity to the junction (marked as Tr) was used in a competition reaction with T1c (in 1:1 molar ratio) to monitor integration specificity (lane 5). Tr contains a shorter (30 nt) 3′ segment following the cleavage site than Tic, so that the integration products can be clearly distinguished. Schematics for the labeled junction substrate (a), the cleavage product (d) and integration products with T1c (b) or Tr (c) are shown on the right. (**D**) J1 integrates selectively into its complementary target substrate (T1c) even in the excess of scrambled target substrates. Competition assays with 2 different scrambled target pools (TsI and TsII), containing a conserved TTAC site and different sets of scrambled sequences in the 8 nt variable region, are shown. The molar ratio of T1c:TsI or T1c:TsII is indicated above the gel. Positions of the J1 substrate (a), cleavage (d) and strand transfer products with T1c (b) or TsI/TsII (c) in the sequencing gel are indicated by arrows.

One remarkable feature of the TnpA/LE29/T6’ structure is that the 5′-end of T6’ is located near the 3′ base of the IP_L_ stem loop in LE29 (Figure 4A). This suggested that introducing additional base pairing interactions at this transposon/target interface might provide a strategy for increasing target site specificity. Therefore, we designed transposon sequences including specific 8 nt long sequences at positions +44 to +51 in LE and corresponding target substrates with a complementary 8 nt sequence upstream of C_T_ (Figure 4B). The region of extended complementarity in the target was placed 3 nt apart from C_T_ to provide flexibility for optimal interaction. The 3 nt linker size was chosen as it best supported TnpA cleavage in our initial tests. Moreover, the triplet-forming A^+42^ base at the 3′ end of LE was mutated to T, to minimize steric constrains while maintaining the triplet interaction.

We then assayed TnpA-mediated cleavage and strand exchange activities of these engineered transposon sequences *in vitro* as previously described (16), which showed that these modified elements were as competent as the wild type element in performing all transposition steps *in vitro*, including LE and RE cleavage, generation of a RE-LE transposon junction and insertion of this junction into a target substrate (Supplementary Figure S3).

To investigate target site specificity, we then analyzed the integration activities of engineered transposon junctions (Ji, with ‘i’ indicating a specific variant number) into complementary targets (Tic) *in vitro* on sequencing PAGE. Several sequence pairs were tested and two representative examples, J1/T1c and J2/T2c, are shown in Figure 4C and Supplementary Figure S4, respectively. The modified transposon junctions integrated efficiently into their complementary target, as shown by the specific formation of strand transfer products between Ji and Tic in all cases (lane 3 in both Figure 4C and Supplementary Figure S4). Integration reactions including equimolar concentrations of the complementary target and a random target with no extra complementarity to the junction substrate beyond the canonical TTAC (C_T_), showed an explicit preference for integration into the complementary target (lane 5 in Figure 4C and in Supplementary Figure S4). The preferential selection of targets with the extra complementarity region was also clearly observed in the presence of two different pools of random targets containing scrambled sequences in the 8 nt variable region (TsI and TsII; see Figure 4B) at various Tic:Ts concentration ratios (Figure 4D, lanes 6–15). In fact, J1 integrated predominantly into its complementary target (T1c), even in the presence of 20-fold molar excess of random target substrates (lanes 9 and 14 in Figure 4D).

**Figure 5.**
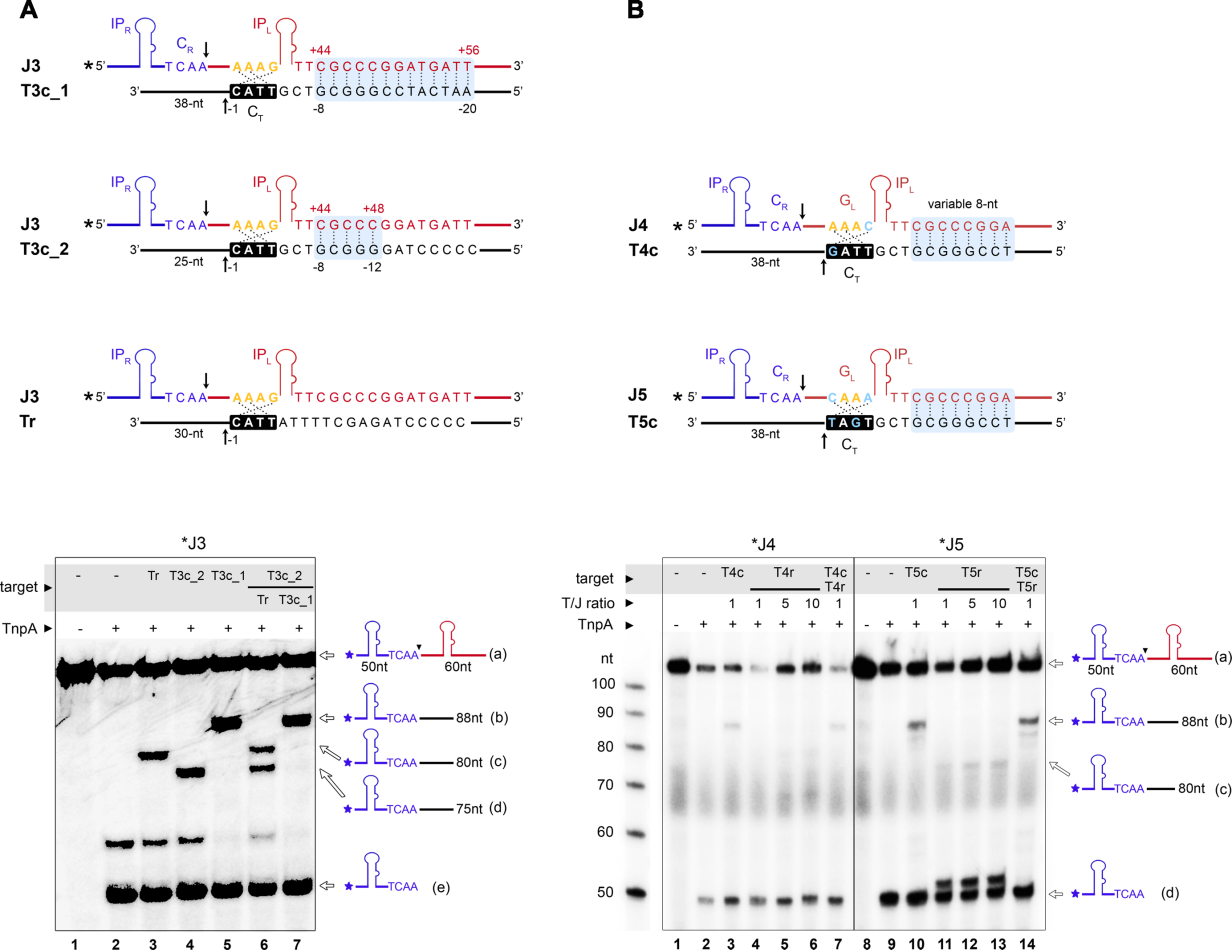
IS*608* integration specificity can be enhanced to 17 nt sites and retargeted to non-native target sites. (**A**) Integration reactions with a junction and target pair engineered to form 13 additional base pairs (see light blue shade in the scheme; J3 and T3c_1) are shown on sequencing PAGE (bottom). J3 integration to T3c_1 was compared with a target containing only 5 nt complementarity in the variable region (light blue; T3c_2) or a random target (Tr) maintaining only the G_L_/C_T_ interaction. Target substrates contain various 3′ segments following the cleavage site to distinguish integration products. In competition reactions (lanes 6, 7), targets were combined in 1:1 molar ratio. Bands corresponding to the substrates and products are indicated on the right. (**B**) IS*608* targeting to integration sites with alternative C_T_ sequences. Two different sets of junction/target complementary pairs with mutations in G_L_ and C_T_ were designed (J4/T4c and J5/T5c), as shown on the top. Light-blue shade highlights the complementary regions and arrow marks the cleavage positions. Reaction products obtained with 5′ ^32^P- labeled J4 and J5 junctions and unlabeled targets were analyzed on a sequencing gel (bottom). Random targets T4r and T5r, containing the same C_T_ sequence as in T4c and T5c, respectively, with a random sequence in the 8 nt variable region were used as control. T4r and T5r contain a shorter (30 nt) 3′ segment following the cleavage site. Integration in T4r or T5r is very inefficient even in large excess of the target substrate (lanes 4–6 and 11–13), whereas T4c and T5c produce more product (lanes 3 and 10) and compete favourably with the random targets (lanes 7 and 14). Substrates and products are shown schematically on the right.

To further explore the scope of our targeting strategy, we tested the ability of IS*608* to select even more specific targets by further increasing the region of base complementarity. Representative data for a junction/target pair with a 13 bp complementary region in addition to the G_L_/C_T_ interaction (J3/T3c_1) are shown in Figure 5A. Integration of J3 to T3c_1 (lane 5) was compared with integration to a random target (Tr, which contains only G_L_/C_T_ complementarity, lane 3) and to a target containing 5 complementary bases in addition to the C_T_ site (T3c_2, lane 4). Remarkably, while the 5 bp long complementarity did not enable efficient selection of T3c_2 over Tr (lane 6), integration was exclusively directed to T3c_1 in the presence of an equimolar amount of T3c_2 (lane 7). These results provide proof of concept for specific targeting of engineered IS*608* transposons to selected 12–17 nt long sequences, with 4 nt defined by the native G_L_/C_T_ interaction and extra 8–13 nt defined by engineered extended complementarity.

### Extended LE/target recognition can be combined with targeting of altered C_T_ sequences

It was previously demonstrated that IS*608* insertion can be redirected to alternative tetranucleotide target sequences by mutating the transposon guide sequence (16). Although engineered transposons were less efficient, they were very specific for integration into the intended sites. Therefore, we analyzed the potential of combining the previous C_T_ resetting strategy with our new extended target recognition method for two different junction/target pairs, J4/T4c and J5/T5c. These substrates contain the same 8 bp extended complementary region as J1/T1c, but with one or two G_L_–C_T_ base pairs also modified (Figure 5B). We assayed TnpA-mediated cleavage and integration activity with these substrates, including competition reactions with random targets T4r and T5r (containing the same C_T_ as in T4c and T5c, respectively, but without extended complementarity to LE). Integration products with J4/T4r and J5/T5r substrate pairs were not detected, even with 10-fold excess of the random target (Figure 5B, lanes 4–6 and 11–13), in agreement with the previously observed decrease in activity with redirected G_L_/C_T_ sites (16). Interestingly, integration activity was greatly enhanced by introduction of the extra 8 bp complementary sequence in the engineered J4/T4c and J5/T5c pairs (lanes 3 and 10), indicating that extended base pairing with the target can rescue transposon integration. The extended complementary target sites were also preferentially chosen in competition experiments (Figure 5B, lanes 7 and 14).

### DNA secondary structure limits target site specificity of the native IS*608* element

**Figure 6.**
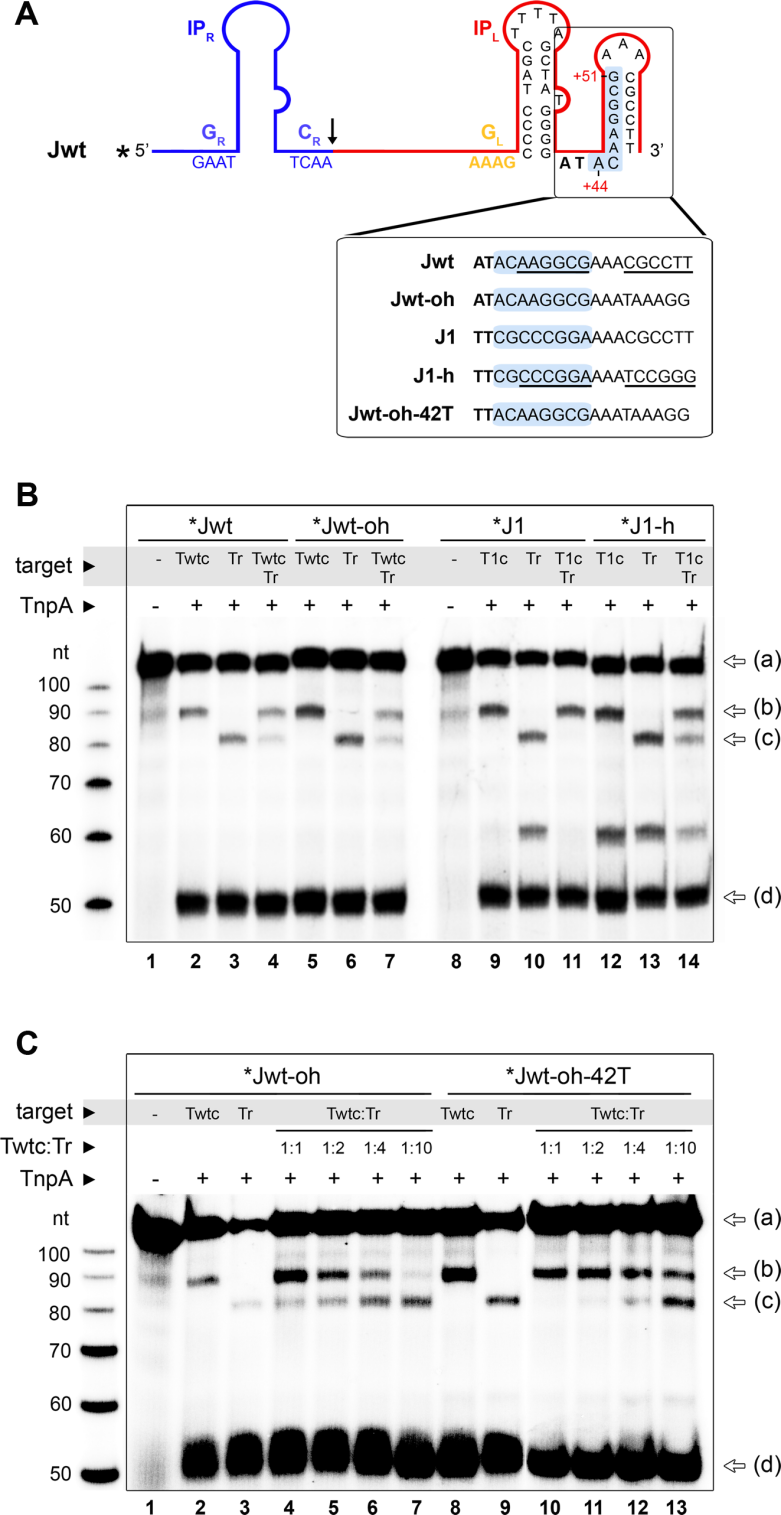
Secondary structure of the IS*608* LE limits target specificity. (**A**) Predicted secondary structure of the wild type IS*608* junction (Jwt) depicts an additional hairpin downstream of IP_L_, which includes the positions used for extended retargeting (highlighted in light blue). To test the role of this hairpin, Jwt and J1 were engineered to disrupt and introduce, base pair complementarity within the second hairpin, respectively (bottom insert). (**B**) Integration activity of radiolabeled Jwt-oh and J1-h junction substrates with unlabeled target oligos containing a complementary (Twtc/T1c) or random (Tr) sequence in the 8 nt variable region used for retargeting. For competition reactions, Twtc/T1c and Tr were combined in 1:1 molar ratio (lanes 4, 7, 11, 14). Reaction substrates and products (identified by arrows on the right, as in Figure 4) were separated on a sequencing PAGE. (**C**) A variant of Jwt-oh including a mutation of A^+42^ to T (Jwt-oh-42T) was analyzed in combination with different Twtc:Tr ratios, as indicated.

Our finding that IS*608* can be targeted to long specific sites by base pairing with the target led us to ask what limits target site specificity of the wild type element. Early analysis of the wild type IS*608* sequence (21) implicated an additional stem-loop structure at the 3′ end of the subterminal IP_L_ involving nucleotide positions +46 to +60 (Figure 6A). This hairpin spans the bases used for extended targeting in our engineered junctions (+44 to +51) so that it could block them from extended pairing with the target DNA. To test the role of this DNA structure, we examined integration of the wild type junction (Jwt) into a complementary target (Twtc), using Jwt with and without the ability to form the 3′ hairpin (Jwt and Jwt-oh, respectively). Jwt-oh (Jwt with open hairpin) was modified in positions +55 to +60 to disrupt the 3′ hairpin structure. Furthermore, we mutated the same positions in the J1 junction to create a 3′ stem-loop (J1-h) (Figure 6A). Selection of each complementary target was evaluated in the presence of a random target as before. Whereas disruption of the second LE hairpin in Jwt-oh did not trigger a marked increase in Twtc selection compared to Jwt (Figure 6B, lanes 4 and 7), introduction of such hairpin in J1 resulted in a clearly reduced specificity for integration into T1c (Figure 6B, lanes 11 and 14). This suggests that the 3′ hairpin may help to prevent complementary base pairing, but some other factor is also involved. Thus, we next evaluated the effect of mutating the triplet-forming A^+42^ in Jwt-oh to a T (Jwt-oh-42T), as was previously done for J1 and J2 in our retargeting strategy (Figure 6C). This mutation to Jwt-oh-42T resulted in an increased efficiency for integration to Twtc, as well as in a significant increase in the selection of Twtc over Tr. Overall, these results show that specific secondary structure features in the LE, including the small hairpin and a bulky triplet-forming base 3′ of the IP, together regulate IS*608* integration efficiency and restrict its ability to form extended base pairings with target substrates.

## DISCUSSION

Target site selection of transposons is a crucial aspect for their genetic engineering and gene delivery applications, yet it is complex and generally poorly understood (24). Most transposons display little sequence specificity for target site selection and choose their insertion sites based on loose sequence constraints, distinctive DNA topology, chromatin state or by association with specific trans-acting factors (25–27). Insertion sequences from the IS*200*/IS*605* family integrate into specific tetra- or pentanucleotide sequences and their integration can be redirected to desired non-native target sites by mutating the transposon sequence (16). Based on our crystallographic structure of the IS*608* target capture complex with fully assembled active site in a pre-integration state, we now elucidate critical features of IS*608* target recognition and develop a strategy to direct integration to long target sequences in a programmable fashion.

The use of an intact target substrate together with Ca^2+^ ions to mimic the divalent metal ion cofactor without supporting cleavage, allowed us to visualize the conformation of the complete IS*608* TnpA active site precisely aligned for target cleavage and transposon integration. The active site includes the metal ion cofactor, the intact nucleophile tyrosine and the scissile phosphate group of the cytosine nucleotide downstream of the cleavage site. The active site geometry and metal ion coordination in the IS*608* TnpA/LE/target complex closely resemble the previously determined *D. radiodurans* IS*Dra2* target capture complex (22), indicating that the chemistry of DNA cleavage and ligation is conserved within the IS*200*/IS*605* transposon family. Furthermore, the active site assembly in the IS*608* TnpA/LE/target complex and the post-cleavage TnpA/RE35 complex (14) are also highly similar (Supplementary Figure S2B), which implies that the transition from the pre-cleavage to the post-cleavage state does not require major conformational rearrangements.

Notably, the C^+1^ base downstream of the cleavage position is turned towards the LE IP in the active pre-cleavage conformation, in a similar arrangement to that observed in the IS*Dra2* structures (22). However, while in IS*Dra2* G^+1^ base pairs with C^+15^, in our IS*608* structure C^+1^ does not form specific interactions with the structurally equivalent A^+18^ in G_L_. Instead, its position is stabilized by non-specific stacking interactions with aromatic amino acids on the protein surface. This C^+1^ binding pocket is fairly tight, clearly disfavoring the binding of bulkier purine bases in the active conformation. In agreement, our biochemical experiments indicated a strong preference for cleavage at TTAC targets followed by C, especially relative to purine bases (A or G) in this position (see Figure 3C). These results are also consistent with previous *in vivo* data, where ∼70% of IS*608* integrations were observed at TTAC|C sites in an F plasmid derivative in *E. coli* (12). Remarkably, integration of IS*Dra2* is even more selective with respect to the identity of the base that follows the cleavage site, as it integrates with ∼90% specificity to TTGAT|G in various *D. radiodurans* strains (28). Altogether, these data show that the identity of the nucleotide immediately after the cleavage site in the target DNA affects integration efficiency and contributes to the target choice of IS*200*/IS*605* family of transposons. However, the selectivity at this position is less stringent than within the core C_T_ target sequence and exploits various mechanisms relying on highly specific base pairing or steric constraints in a snug pocket in TnpA.

Another remarkable feature of the IS*608* target capture complex is that the two base pairs (A^+16^:T^−3^ and A^+17^:T^−4^) that participate in transposon-target recognition form base triplets with bases at the 3′ extension of the LE IP (T^+43^ and A^+42^, respectively). These base triplets strengthen the interaction between LE and the target DNA and likely help stabilize the architecture of the active synaptic complex. Similar base triplets are also formed on the RE, where they help establish a compact DNA structure and guide the RE cleavage site into the active site of TnpA. Replacement of the A^+16^:T^−3^ and A^+17^:T^−4^ base pairs was previously shown to significantly decrease integration efficiency (16). Furthermore, the presence of A^+42^ and T^+43^ in IS*608* LE is essential for the assembly of a stable synaptic complex (15).

Based on the structural insights into the architecture of the complete target capture complex, we developed a strategy to direct IS*608* integration into longer arbitrarily chosen target sequences that have the potential to be unique in a genomic context. By mutating the transposon left end downstream of IP_L_ to a sequence that is complementary to the desired target DNA sequence, we generated a new specific interaction network that allows the transposon to recognize various 12 nt and 17 nt long complementary target sites. These engineered transposons efficiently target the intended DNA sites without impairing any of the transposition steps *in vitro*. Importantly, this extended targeting strategy can be efficiently combined with previously demonstrated transposon engineering approaches that allow resetting of the core C_T_ target site (16). In fact, our engineered transposons even rescue the low integration frequency observed with some non-native target tetranucleotides.

Interestingly, efficient retargeting by extended target/LE base-pairing also required mutating A^+42^ to T and disrupting a small second hairpin loop downstream of IP_L_ (Figure 6C). In the crystal structure, A^+42^ (at the 3′ end of IP_L_) forms a non-canonical base pair with A^+17^ from G_L_, which in turn interacts with T^−4^ together creating a base triplet. As T-AT base triplets have been reported to be more stable than A-AT triplets (29), we speculate that introduction of a T in place of A^+42^ further stabilizes this interaction. The A to T replacement probably also removes steric constraints that may restrict extended LE/target base pairing. On the other hand, the second hairpin at the LE (positions +46 to +60) was shown to have a minor role in the stabilization of TnpA binding to LE (15) and it is dispensable for the *in vitro* cleavage or strand transfer activities (Figure 6). However, the presence of this second hairpin buries the nucleotides we used for extended target recognition in the ssDNA intermediate, thus preventing them from interacting with the target DNA site and reducing the efficiency of retargeting. Such second hairpin is also present on the 3′ side of the main subterminal palindrome in the left end of other members of the IS*200*/IS*605* family, such as IS*Dra2* and IS*605* from *H. pylori*, suggesting that it may play a general regulatory role in IS*200*/IS*605* transposition. We believe that the second LE hairpin might serve as a natural constraint for sustaining a fairly loose specificity in target choice, and together with the suboptimal base triplet 3′ of IP_L_ may provide a double-layered mechanism to prohibit overly specific integration. Extensive target specificity would namely reduce the number of accessible genomic target sites, providing a disadvantage for transposon dissemination and evolution in the natural setting (24,30).

The targeting strategy we propose here presents various attractive features for genetic applications. It is based on the simplest transposon known to date and only requires short DNA sequences and a small protein that is easy to produce, store and use. It offers programmable targeting to selected DNA sites by simply modifying the transposon DNA sequence to create simple DNA-DNA base pairing with the target site. The ease and efficacy of such targeting strategy is best demonstrated by the CRISPR/Cas systems, which rely on base pairing between a suitably designed guide RNA and the target DNA to execute specific cleavage at any chosen genomic site. The IS*608* system uses stable DNA guides and directly integrates its cargo into a target DNA without double-strand breaks. However, further experiments will be necessary to establish the efficient use of the IS*608*-based targeting strategy in a genomic context *in vivo* in different cellular backgrounds. IS*608* has been demonstrated to move in its native *H. pylori* as well as in the heterologous host *E. coli*. If efficient retargeting can be achieved, engineered IS*608* transposons may enable site-specific modification of diverse bacterial hosts. This would be particularly beneficial in *H. pylori*, given its impact in gastric infections and cancer and the lack of efficient genome engineering tools in these bacteria. The functionality of IS*608* has not yet been demonstrated in eukaryotic systems, but some prokaryotic IS sequences (IS*607*-like) have been identified in eukaryotic genomes (31), raising the possibility that some elements could be active in eukaryotes. In case IS*608* were active in eukaryotic cells, our targeting strategy would open up new possibilities for genome engineering broadly. Nevertheless, the demonstrated ability of IS*608* to precisely excise and integrate DNA fragments or genes at highly specific positions without genetic scars *in vitro*, also offers attractive technological applications. For instance, it may be used to specifically detect single-stranded intermediates of transposition, replication or conjugation, or for cloning long or repetitive DNA inserts effectively at flexible positions (without constraints on primers or restriction sites) in plasmid DNA.

## DATA AVAILABILITY

Atomic coordinates and structure factors for the reported crystal structure have been deposited with the Protein Data Bank under accession number 6FI8.

## Supplementary Material

Supplementary Data
