# Supplementary material for: Targeting IS608 transposon integration to highly specific sequences by structure-based transposon engineering
Source: Nucleic Acids Res. 2018 Apr 9;46(8):4152–63. doi: 10.1093/nar/gky235 (PMC5934647; doi:10.1093/nar/gky235)
Supplement: Supplementary Data [file gky235_supp.pdf]

Supplementary Material

Targeting IS*608* transposon integration to highly specific sequences by structure-based transposon engineering

Natalia Rosalía Morero[1], Cecilia Zuliani[1,†], Banushree Kumar[1,†], Aleksandra Bebel[1], Sachi Okamoto[1], Catherine Guynet[2], Alison Burgess Hickman[3], Michael Chandler[2], Fred Dyda[3,*], and Orsolya Barabas[1,*]

[1] Structural and Computational Biology Unit, European Molecular Biology Laboratory, Heidelberg, 69117, Germany
[2] Laboratoire de Microbiologie et Génétique Moléculaires, Centre National de la Recherche Scientifique, Toulouse Cedex, 31062, France
[3] Laboratory of Molecular Biology, National Institute of Diabetes and Digestive and Kidney Diseases, National Institutes of Health, Bethesda, MD 20892, USA

[†] These authors contributed equally to this work.

* To whom correspondence should be addressed. Tel: +49-6221-3878626; Fax: +49 6221 387 8519; Email: barabas@embl.de
Correspondence may also be addressed to Fred Dyda. Email: fred.dyda@nih.gov

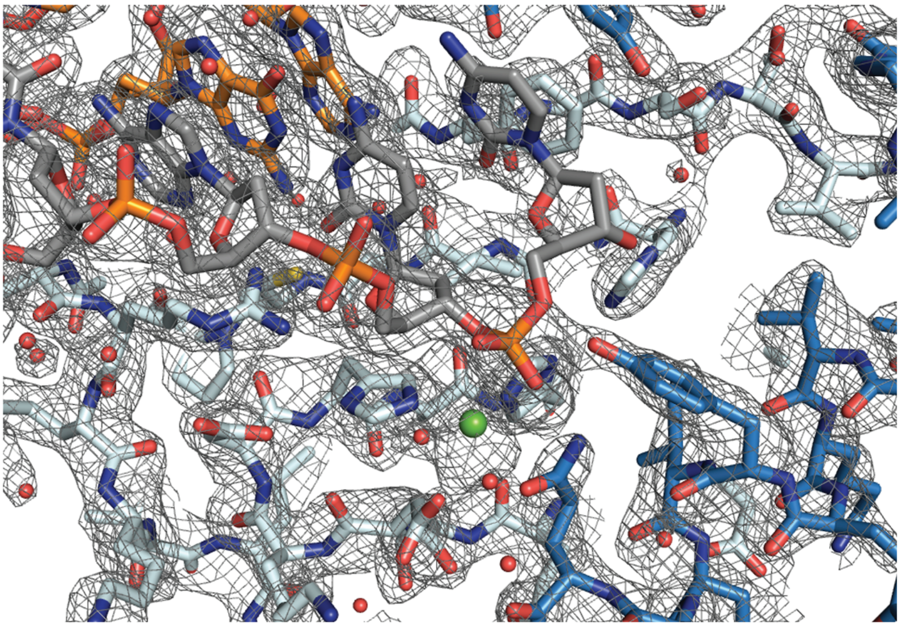

Supplementary Figure 1. 2Fo-Fc composite omit electron density map and final molecular model for the IS*608* TnpA/LE29/T6' complex. The map (black mesh) shows the region of the active site of one target capture complex, contoured at 1.1 sigma level. Protein (in two shades of blue) and DNA (T6' in grey and LE29 in orange) residues are shown as sticks with atomic colouring; water molecules and Ca^{2+} ions are shown as red and green spheres, respectively.

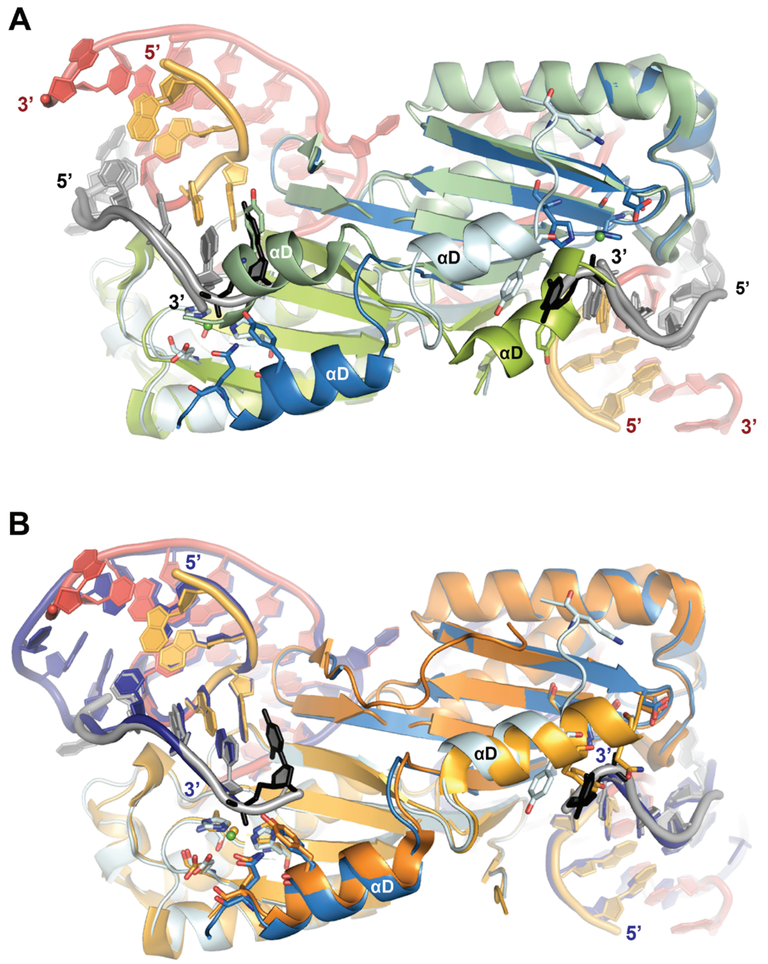

Supplementary Figure 2. Comparison of IS*608* TnpA – DNA complex structures. (A) Structural alignment of the TnpA/LE29/T6’ target capture complex structure (protein in blue/light blue cartoon) with the previously determined post-cleavage TnpA/LE26/T6 structure (green/lime) (12). LE molecules are shown in red (with G_L in orange) and target oligos in grey (with C^{+1} from T6’ highlighted in black). Catalytic protein residues are shown in sticks representation with atomic colouring. Green balls are Ca^{2+} ions. Helix αD and the nucleophile tyrosine move from an inactive conformation in the TnpA/LE26/T6 complex to an active position in the fully assembled TnpA/LE29/T6’ complex (compare green and blue molecules). (B) Structural alignment of the TnpA/LE29/T6’ structure (protein in blue/light blue, LE DNA in red/orange, T6’ DNA in grey, Ca^{2+} ions in green) with the TnpA/RE35 (12) complex containing the complete right transposon end (protein in dark/light orange, DNA in dark blue, Mn^{2+} ions in orange). The protein conformation in both structures is remarkably similar.

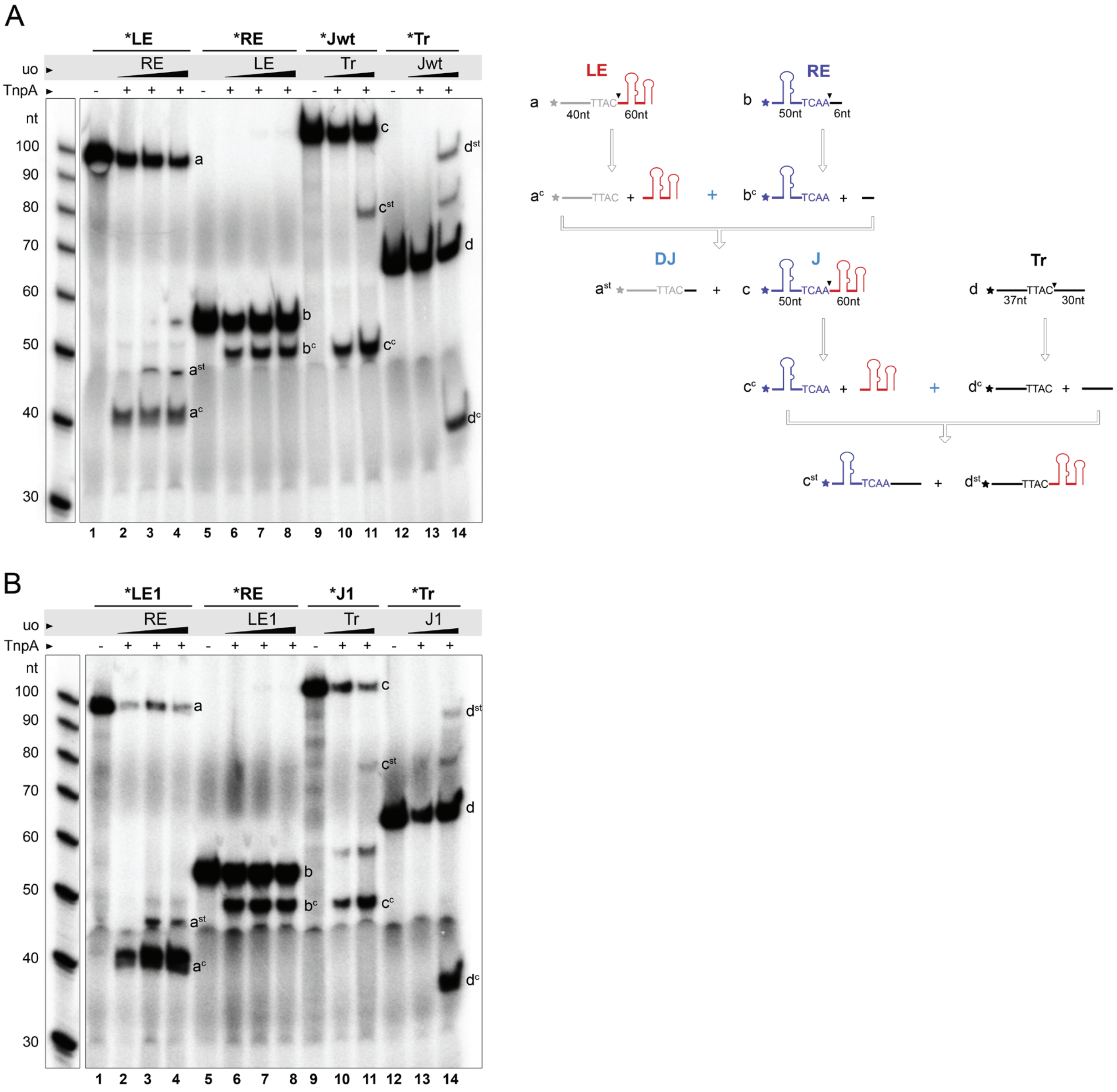

Supplementary Figure 3. The engineered IS*608* transposon with extended LE/target complementarity is fully competent for transposition. (A) Sequencing PAGE gel showing generation of transposition intermediates and products with the wild type (wt) IS*608* element *in vitro*. Reactions were performed with 5' ^{32}P labeled substrates (marked with asterisk) and unlabeled oligos (uo) at increasing concentrations, as indicated. Schematic representation of substrates and products are shown on the right. The following reaction steps were assayed: cleavage of *LE (generating product a^{c}) and strand transfer from cleaved *LE to RE resulting in formation of the donor joint (DJ) (a^{st}; lanes 2-4); cleavage of *RE (with product b^{c}) and strand transfer from cleaved *RE to LE and formation of the transposon junction (J) (labeled c; lanes 6-8); cleavage of the *J transposon junction (creating product c^{c}) and strand transfer to unlabeled Tr (c^{st}; lanes 10-11), cleavage of the *Tr target (producing d^{c}) and strand transfer to Jwt (d^{st}; lanes 13-14). (B) All transposition intermediates and products are also generated with engineered LE1 and J1 sequences. Cleavage and strand transfer reactions were performed as for the wild type transposon in A and corresponding products are marked with the same letter code.

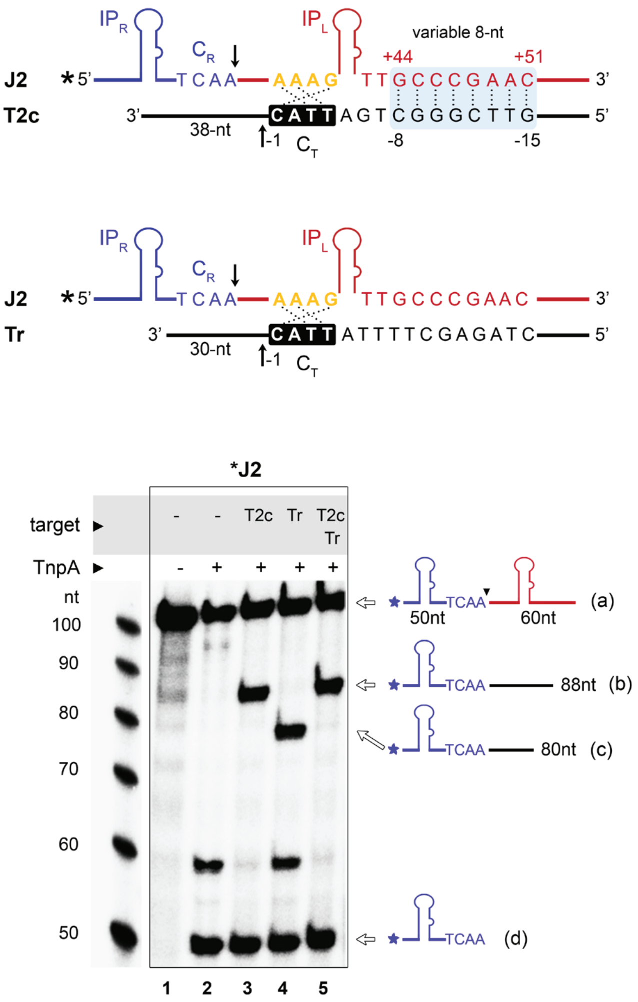

Supplementary Figure 4. Complementary LE/target base pairing allows to target IS*608* to diverse integration sites. Specific targeting of a second transposon variant (J2) to its complementary target (T2c) is shown as in Figure 4. Design of the IS*608* transposon junction and target substrates is shown on the top, with the variable 8 bp complementary region highlighted in light blue. ^{32}P radioisotope labeling is indicated by an asterisk. Upon target cleavage and integration (at the arrow), the radiolabeled 5' segment of the junction upstream of the cleavage site (50 nt) is attached to the 3' segment of the target (38 nt). J2 cleavage and integration is monitored on sequencing DNA PAGE gel (bottom). A random target substrate containing a TTAC site with no additional complementarity (Tr) was used to assess integration specificity (lane 5, with T2c and Tr in 1:1 molar ratio). Tr contains a 30 nt 3' segment following the cleavage site and can be clearly distinguished from T2c on the gel. Schematics for the substrates and products are shown on the right.

Supplementary Table 1. Oligonucleotides

	Sequence (5 ’to 3’)
Crystallization	
LE29	AAAGCCCCTAGCTTTTAGCTATGGGGATA
T6’	ATTACC
Activity Assays	
LE	CGGGCTGCAGGAATTCGATTTGCGCTAGTGCAAAAATTACCAAAACTAACGCCTTAAAGCCCCTAGCTTTTAGCTATGGGGATACAAGGCGAAACGCCTT
LE1	CGGGCTGCAGGAATTCGATTTGCGCTAGTGCAAAAATTACCAAAACTAACGCCTTAAAGCCCCTAGCTTTTAGCTATGGGGTTCGCCCGGAAAACGCCTT
RE	CTCATGCTTTAGCTAGAATCCCCTAGCTTTAGCTATGGGGAGTATGTCAACAAATT
Jwt	CTCATGCTTTAGCTAGAATCCCCTAGCTTTAGCTATGGGGAGTATGTCAACAAAACTAACGCCTTAAAGCCCCTAGCTTTTAGCTATGGGGATACAAGGCGAAACGCCTT
Jwt-oh	CTCATGCTTTAGCTAGAATCCCCTAGCTTTAGCTATGGGGAGTATGTCAACAAAACTAACGCCTTAAAGCCCCTAGCTTTTAGCTATGGGGATACAAGGCGAAATAAAGG
Jwt-oh-42T	CTCATGCTTTAGCTAGAATCCCCTAGCTTTAGCTATGGGGAGTATGTCAACAAAACTAACGCCTTAAAGCCCCTAGCTTTTAGCTATGGGGTTACAAGGCGAAATAAAGG
J1	CTCATGCTTTAGCTAGAATCCCCTAGCTTTAGCTATGGGGAGTATGTCAACAAAACTAACGCCTTAAAGCCCCTAGCTTTTAGCTATGGGGTTCGCCCGGAAAACGCCTT
J1-h	CTCATGCTTTAGCTAGAATCCCCTAGCTTTAGCTATGGGGAGTATGTCAACAAAACTAACGCCTTAAAGCCCCTAGCTTTTAGCTATGGGGTTCGCCCGGAAAATCCGGG
J2	CTCATGCTTTAGCTAGAATCCCCTAGCTTTAGCTATGGGGAGTATGTCAACAAAACTAACGCCTTAAAGCCCCTAGCTTTTAGCTATGGGGTTGCCCGAACAAACGCCTT
J3	CTCATGCTTTAGCTAGAATCCCCTAGCTTTAGCTATGGGGAGTATGTCAACAAAACTAACGCCTTAAAGCCCCTAGCTTTTAGCTATGGGGTTCGCCCGGATGATTCCTT
J4	CTCATGCTTTAGCTAGAATCCCCTAGCTTTAGCTATGGGGAGTATGTCAACAAAACTAACGCCTTAAACCCCCTAGCTTTTAGCTATGGGGTTCGCCCGGAAAACGCCTT
J5	CTCATGCTTTAGCTAGAATCCCCTAGCTTTAGCTATGGGGAGTATGTCAACAAAACTAACGCCTTCAAACCCCTAGCTTTTAGCTATGGGGTTCGCCCGGAAAACGCCTT
Tr	CCGATGATGAGGAACCCCCCCCTAGAGCTTTTATTACTGATGATGAGGAACCCCCCCCTAGTGATGA
Twtc	CCGATGATGAGGAACCCCCCCCCGCCTTGTTTATTACTGATGATGAGGAACCCCCCCCTAGTGATGATGAGGATT
T1c	CCGATGATGAGGAACCCCCCCCTCCGGGCGTCGTTACTGATGATGAGGAACCCCCCCCTAGTGATGATGAGGATT
T2c	CCGATGATGAGGAACCCCCCCCGTTCGGGCTGATTACTGATGATGAGGAACCCCCCCCTAGTGATGATGAGGATT
T3c_1	CCGATGATGAGGAACCCAATCATCCGGGCGTCGTTACTGATGATGAGGAACCCCCCCCTAGTGATGATGAGGATT
T3c_2	CCGATGATGAGGAACCCCCCCCTAGGGGCGTCGTTACTGATGATGAGGAACCCCCCCCTAGT
T4c	CCGATGATGAGGAACCCCCCCCTCCGGGCGTCGTTAGTGATGATGAGGAACCCCCCCCTAGTGATGATGAGGATT
T5c	CCGATGATGAGGAACCCCCCCCTCCGGGCGTCGTGATTGATGATGAGGAACCCCCCCCTAGTGATGATGAGGATT
T4r	CCGATGATGAGGAACCCCCCCCTAGAGCTTTTATTAGTGATGATGAGGAACCCCCCCCTAGTGATGA
T5r	CCGATGATGAGGAACCCCCCCCTAGAGCTTTTATGATTGATGATGAGGAACCCCCCCCTAGTGATGA
